# GLYX-13 Ameliorates Schizophrenia-Like Phenotype Induced by MK-801 in Mice: Role of Hippocampal NR2B and DISC1

**DOI:** 10.3389/fnmol.2018.00121

**Published:** 2018-04-11

**Authors:** Dongsheng Zhou, Dan Lv, Zhen Wang, Yanhua Zhang, Zhongming Chen, Chuang Wang

**Affiliations:** ^1^Ningbo Kangning Hospital, Ningbo, China; ^2^Ningbo Key Laboratory of Behavioral Neuroscience, School of Medicine, Ningbo University, Ningbo, China; ^3^Zhejiang Provincial Key Laboratory of Pathophysiology, School of Medicine, Ningbo University, Ningbo, China; ^4^Department of Physiology and Pharmacology, School of Medicine, Ningbo University, Ningbo, China; ^5^Key Laboratory for Receptor Research, Shanghai Institute of Materia Medica, Chinese Academy of Sciences, Shanghai, China

**Keywords:** GLYX-13, disrupted-in-schizophrenia 1, *N*-methyl-D-aspartate receptor, *N*-methyl D-aspartate receptor subtype 2B, schizophrenia

## Abstract

**Background:** Evidence supports that the hypofunction of *N*-methyl-D-aspartate receptor (NMDAR) and downregulation of disrupted-in-schizophrenia 1 (DISC1) contribute to the pathophysiology of schizophrenia. *N*-Methyl D-aspartate receptor subtype 2B (NR2B)-containing NMDAR are associated with cognitive dysfunction in schizophrenia. GLYX-13 is an NMDAR glycine-site functional partial agonist and cognitive enhancer that does not induce psychotomimetic side effects. However, it remains unclear whether NR2B plays a critical role in the GLYX-13-induced alleviation of schizophrenia-like behaviors in mice.

**Methods:** The effect of GLYX-13 was tested by observing changes in locomotor activity, novel object recognition ability, and prepulse inhibition (PPI) induced by dizocilpine (known as MK-801) in mice. Lentivirus-mediated NR2B knockdown in the hippocampus was assessed to confirm the role of NR2B in GLYX-13 pathophysiology, using Western blots and immunohistochemistry.

**Results:** The systemic administration of GLYX-13 (0.5 and 1 mg/kg, i.p.) ameliorates MK-801 (0.5 mg/kg, i.p.)-induced hyperlocomotion, deficits in memory, and PPI in mice. Additionally, GLYX-13 normalized the MK-801-induced alterations in signaling molecules, including NR2B and DISC1 in the hippocampus. Furthermore, we found that NR2B knockdown produced memory and PPI deficits without any changes in locomotor activity. Notably, DISC1 levels significantly decreased by NR2B knockdown. However, the effective dose of GLYX-13 did not alleviate the memory and PPI dysfunctions or downregulation of DISC1 induced by NR2B knockdown.

**Conclusion:** Our results suggest GLYX-13 as a candidate for schizophrenia treatment, and NR2B and DISC1 in the hippocampus may account for the molecular mechanisms of GLYX-13.

## Introduction

Schizophrenia, a chronic and degenerative disease, has an overall lifetime risk of 1%. However, the etiologies of schizophrenia remain unclear. Recent studies have suggested that *N*-methyl-D-aspartate receptor (NMDAR) hypofunction contributes to the negative symptoms and cognitive impairments observed in schizophrenia (Gilmour et al., 2012; Jadi et al., 2016; Dauvermann et al., 2017). This putatively occurs due to dysfunction of glutamate transmission at NMDARs in this disease. Based on these findings, it has been postulated that hippocampal NMDAR may underlie some schizophrenia-like phenotypes elicited by NMDAR blockers (Geddes et al., 2014; Leung and Ma, 2017) and may become the candidate target for drug development.

Positive allosteric modulators represent an alternative approach for the reversal of NMDAR hypofunction (Iwata et al., 2015; Yao and Zhou, 2017). Recent drug discovery efforts have identified GLYX-13, an NMDAR modulator with glycine-site partial agonist properties, to significantly enhance learning and synaptic plasticity (Moskal et al., 2005, 2014; Rodriguez et al., 2016). Recently, GLYX-13 was shown to ameliorate subchronic phencyclidine- and ketamine-induced declarative memory deficits (Rajagopal et al., 2016), indicating its cognition-enhancing properties in schizophrenia. However, whether GLYX-13 reverses schizophrenia-like behaviors induced by dizocilpine (known as MK-801) remains unclear. We evaluated whether GLYX-13 can ameliorate MK-801-induced psychotomimetic behaviors, such as hyperlocomotion, memory deficits, and sensorimotor gating disruption, in mice.

Interestingly, the administration of MK-801 causes a reduction in disrupted-in-schizophrenia 1 (DISC1) (Ramsey et al., 2011), which is one of the main candidate genes for schizophrenia. Additionally, the lentivirus-mediated exogenous overexpression of DISC1 partially rescues the overextended migration of newborn neurons induced by NMDAR antagonists (Namba et al., 2011), indicating that DISC1 is regulated by NMDAR in the schizophrenia. Notably, NMDAR are composed of seven subunits. Reduced expression of the *N*-methyl D-aspartate receptor subtype 2B (NR2B) subunit has been reported in schizophrenia (Kristiansen et al., 2010; Geddes et al., 2014; Yang et al., 2015; Gulchina et al., 2017). Our current study raises the question of whether NR2B and DISC1 play a critical role in the GLYX-13-induced alleviation of schizophrenia-like symptoms in mice. We used the lentivirus-mediated NR2B knockdown in the hippocampus to identify the contribution of NR2B/DISC1 signaling in GLYX-13-induced alleviation of schizophrenia symptoms.

## Materials and Methods

### Animals

Adult male C57BL/6J mice (age: 2–3 months upon arrival) were reared in the animal facility of Medical School, Ningbo University, China. The experiments were conducted 7 days after the arrival. All mice were housed (five/cage) in a controlled environment at 22 ± 3°C and 60 ± 5% relative humidity under a 12-h light/dark cycle (lights on at 7:00 AM) with *ad libitum* access to food and water, except as mentioned below. All procedures involving animals were conducted following the National Institute of Health Guidelines for the Care and Use of Laboratory Animals (NIH Publications No. 80-23, revised 1996) as well as the European Community Council Directive for the Care and Use of Laboratory Animals as of September 22, 2010 (2010/63/EU). All experiments were approved by the Institutional Animal Care and Use Committee of the Medical School of Ningbo University.

### Drug Administration

The following drugs were used: GLYX-13 trifluoroacetate and MK-801 (Sigma-Aldrich, St. Louis, MO, United States). We diluted MK-801 in 0.9% saline (vehicle 1) and GLYX-13 in 2% Tween 80 in 0.9% saline (vehicle 2). The doses of GLYX-13 and MK-801 used in the present study were based on previous studies (Moskal et al., 2005; Huang et al., 2014) with minor modifications according to our preliminary data. All drugs were freshly prepared. Intraperitoneal (IP) injection of MK-801 and intravenous (IV) injection of GLYX-13 were administered at a volume of 0.1 mL/10 g.

### Anesthesia, Surgery, and Lentiviral Microinjection

Mice were anesthetized in an induction box with 3.5% isoflurane, maintained with continuous administration of 2.5% isoflurane through a nose cone, and placed in a stereotaxic frame. The stereotaxic coordinates for the dentate gyrus (DG) regions of the hippocampus were plotted in accordance with the Paxinos/Franklin mouse atlas (Paxinos and Franklin, 2001). Bilateral hippocampus infusions were performed via a 10-μl Hamilton microsyringe with a 30-gauge needle fitted to the arm of the stereotaxic apparatus. The injection needle was inserted into the dorsal DG (AP, -1.7 mm from the bregma; ML, ±1.8 mm from the midline; DV, -2.0 mm from the dura) on each side. The design and synthesis of the non-targeting control lentiviral vector containing scrambled small interfering RNA (NC siRNA) and NR2B siRNA of mice was conducted according to the Tuschl rule (Elbashir et al., 2002). The siRNA sequences targeting NR2B (5′-AGCUCGUUCCCAAAAGAGCUU-3′ or 3′-UUUCGAGCAAGGGUUUUCUCG-5′) was used. The NCsiRNA was constructed with a similar process (5′-GCACGACUUCUUCAAGUCCUU-3′ or 3′-UUCGUGCUGAAGAAGUUCAGG-5′). Both NR2B-siRNA and green fluorescent protein (GFP)-siRNA were synthesized by Shanghai GenePharma, Co., Ltd. Mice were bilaterally injected with either NC siRNA or NR2B siRNA-encoding lentiviral vector (1 μl/side) into the hippocampus at a rate of 0.2 μl/min using a multi-channel syringe pump (RWD Life Science, Shenzhen, China). The needle was slowly retracted after an additional 5 min to ensure adequate diffusion of the vectors.

### Behavioral Tasks

#### Open-Field Test

Mice were placed into the center of a plexiglass box (50 cm × 50 cm × 39 cm) equally divided into 16 squares allowing free movement, in a brightly lit room. During a 30-min session, we evaluated horizontal locomotor activity, as previously described (Lee et al., 2013). Horizontal locomotor activity was expressed the total distance (in cm) traveled by the mice over a period of 30 min. Animal behavior was recorded and subsequently analyzed using a video-tracking system (Shanghai Mobile Datum Information Technology Company, Shanghai, China).

#### Novel Object Recognition Task

The novel object recognition task (NORT) was performed using the same apparatus as the open-field test (OFT) with slight modifications from a previous study (Hashimoto et al., 2005). During the acquisition trial, mice were allowed to explore two identical objects for 5 min. The recognition index for a familiar object was calculated using the following formula: Recognition index = [Time spent exploring one of the objects/Total time exploring two identical objects] × 100%. Following a 24-h intertrial interval after training, we performed the retention trial. During the retention trial, mice were allowed to explore one familiar object from the acquisition trial and a novel object. The location of the novel object in the retention trial was randomly assigned for each mouse tested using a pseudorandom schedule. Object exploration was defined as licking, sniffing at a distance less than 2 cm, or touching the objects with the nose and/or forepaws. Exploration time (s) for each object in each trial was manually recorded using two stopwatches. If a mouse failed to explore an object longer than one second in both acquisition and retention trials, they were excluded from the analysis. No mice were excluded in the NORT. Sitting on or turning around the objects were not considered exploratory behaviors. The discrimination index for the novel object in the retention trials was calculated with the following formula: Discrimination index = [(time spent exploring the novel object - time spent exploring the familiar object)/total exploration time] × 100%. After each trial, the apparatus and objects were cleaned with 1% ethanol spray.

#### Prepulse Inhibition (PPI)

Startle reactivity was measured to assess sensorimotor gating using SR-LAB startle chambers (San Diego Instruments, San Diego, CA, United States). The enclosures consisted of a Perspex plexiglass cylinder with 40-mm diameter platform and a piezoelectric unit that converted movement into analog signals that were then digitized and recorded by a computer. The platform and mouse enclosure were placed in an illuminated, ventilated, and sound-attenuated chamber. All testing was performed during the dark phase of the light/dark cycle. Animals were acclimatized to the startle chambers during one 30-min session the day before testing. Mice were placed in the startle chambers and exposed to a 5-min acclimatization period (65-dB background noise) before the session started. After the acclimatization period, mice were presented with combinations of startle (120 dB), prepulse pulse (4, 8, or 12 dB over the 65-dB background, followed by 120 dB), and null trials over a 25-min period. The activity was recorded during all trials. Prepulse inhibition (PPI) was calculated as a percentage of the startle response using the following formula:%PPI = [1 – startle amplitude after prepulse – pulse pair/startle amplitude after pulse only] × 100.

### Immunohistochemistry Analysis

Immunohistochemical analyses were performed to quantify NR2B- and DISC1-immunopositive cells in the DG regions of the hippocampus. Brains were dissected and post-fixed in 4% paraformaldehyde (PFA) at 4°C overnight and immersed in 20% sucrose (in 4% PFA) followed by 30% sucrose [in 0.1 M phosphate-buffered saline (PBS)]. Serial coronal sections of the hippocampus (30 μm thick) were collected on a cryostat (Leica, Wetzlar, Germany). Free-floating sections were permeabilized with 0.2% Triton X-100 in PBS for 15 min, blocked with 5% donkey serum (in PBS) for 1 h at room temperature, and incubated with anti-NR2B (1:800, Abcam, ab93610, Cambridge, MA, United States) and anti-DISC1 (1:800; Abcam, Cat#: ab192258, Cambridge, MA, United States) antibodies overnight at 4°C. The next day, all sections were washed in PBS and incubated with fluorescent-labeled secondary antibodies, anti-rabbit conjugated with Alexa Fluor 488 (1:1000; Invitrogen, Cat#: 11034, Carlsbad, CA, United States), and anti-mouse conjugated with Alexa Fluor 594 (1:1000; Invitrogen, Cat#: 21135, Carlsbad, CA, United States) for 1 h at room temperature. DNA (nuclei) was stained with 4′,6-diamidino-2-phenylindole (DAPI) for 15 min, mounted onto slides, and coverslipped with Pro Long Gold Antifade Mountant (Invitrogen). The images were analyzed using a confocal laser-scanning microscope (LSM710, Zeiss, Germany). Six sections per mouse and five mice per group were used to quantify immunopositive cells. Six hippocampal sections were collected every 30 μm. The numbers of NR2B or DISC1-positive cells in the DG regions were quantified in each section. Cell counts were performed blinded to the experimental conditions.

### Western Blot Analysis

Briefly, hippocampual tissue was homogenized in a radioimmunoprecipitation assay lysis buffer [50 mM Tris-HCl, 150 mM NaCl, 1% NP-40, 0.5% sodium deoxycholate, 0.1% sodium dodecyl sulfate (SDS), pH 7.4; Upstate, Temecula, CA, United States] containing protease and phosphatase inhibitors (Pierce Biotechnology, Rockford, IL, United States) then centrifuged at 15,000 × *g* for 30 min. Protein samples (20 μg total protein) were separated by SDS polyacrylamide gel electrophoresis in 8–10% gels and transferred onto polyvinylidene difluoride membranes (0.2 or 0.45 μm). The membranes were incubated with anti-NR2B (1:1000; Abcam, Cat#: ab28373, Cambridge, MA, United States), anti-DISC1 (1: 1000; Abcam, Cat#: ab192258, Cambridge, MA, United States), and anti-GAPDH (1:1000; Millipore, Cat#: ABS16, Temecula, CA, United States) at 4°C overnight. The membranes were then incubated with either Alexa Fluor 700 (for NR2B, Cat#: A21036, Thermo Fisher Scientific, Waltham, MA, United States; and DISC1, Cat#: A21038, Thermo Fisher Scientific, United States)- or Alexa Fluor Plus 800 (for NR2B, Cat#: A32723, Thermo Fisher Scientific, United States; for DISC1 and GAPDH, Cat#: A32735, Thermo Fisher Scientific, Waltham, MA, United States)-conjugated secondary antibodies (1:10000) for 60 min. Target bands were detected and quantified using a fluorescence scanner (Odyssey Infrared Imaging System, LI-COR Biotechnology, Lincoln, NE, United States).

### Statistical Analyses

**FIGURE 1:**
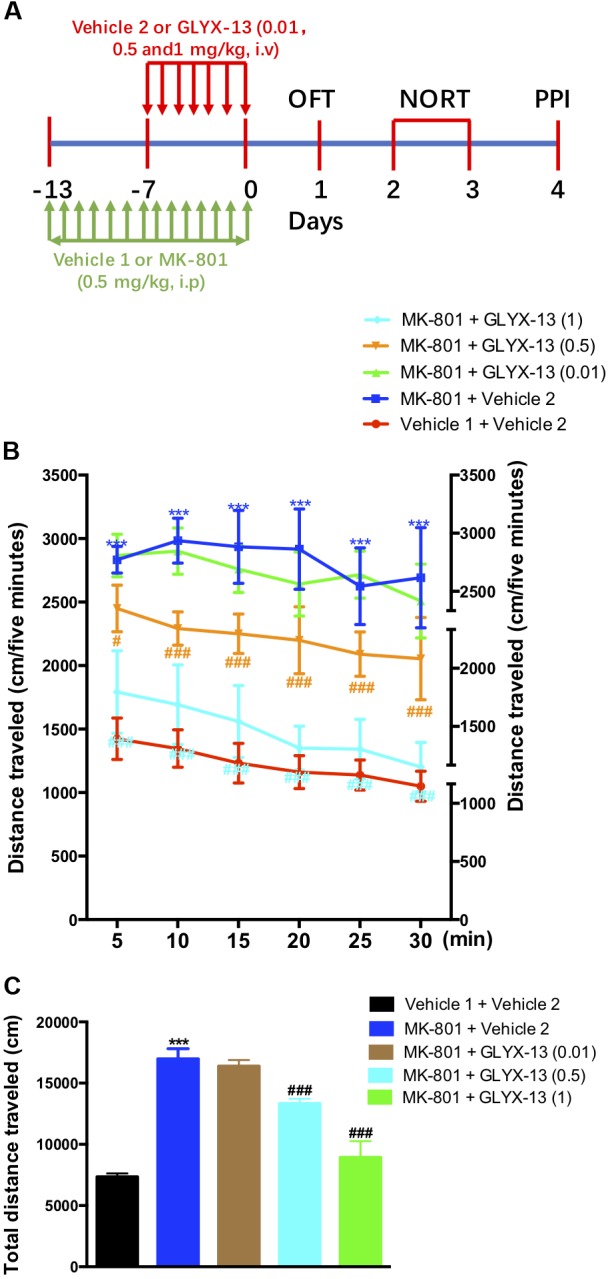
Experimental time line and effects of GLYX-13 on MK-801-induced hyperactivity in the open-field test (OFT). **(A)** Mice received daily administration of vehicle 1, MK-801 (0.5 mg/kg, i.p.), for 14 days. Vehicle 2, GLYX-13 (0.01, 0.5, and 1 mg/kg, i.v.), treatment began on the 7th day after the start of MK-801 administration and continued until the end of the treatment. Twenty-four hours after the last drug treatment animals completed the OFT, novel object recognition task (NORT), and prepulse inhibition (PPI) test between day 1 and day 4. Immediately after the PPI, the hippocampus was removed and processed to assess immunohistochemical changes in NR2B and DISC1 expression, and changes in protein expression by western blotting. **(B,C)** After 14 days of treatment with MK-801, hyperactivity was induced in mice and compared to the control (vehicle 1 and vehicle 2) groups. Horizontal locomotor activity was examined as the distance traveled (cm) in 30 min. The distances traveled in 5 min intervals **(B)** and the total distance traveled in 30 min **(C)** are shown. Data represent the mean ± SEM (*n* = 12 per group; ^∗∗∗^*P* < 0.001, versus the vehicle 1 + vehicle 2-treated group; ^#^*P* < 0.05 and ^###^*P* < 0.001, versus the MK-801 + vehicle 2-treated group).

All measurements were performed by an independent investigator blinded to the experimental conditions. Data are represented as the mean ± standard error of the mean. We used an unpaired Student’s *t*-test (**Figure 5D**) a one-way or repeated-measures two-way (**Figures 1B**, **6B**) analysis of variance (ANOVA) for more than two groups, followed by Newman–Keuls *post hoc* test using GraphPad Prism for PC (Version 6.0, GraphPad, La Jolla, CA, United States) where *P* < 0.05 (two-tailed) was considered significant.

## Results

### Effects of GLYX-13 on MK-801-Induced Hyperlocomotion

The experimental schedule was shown in the **Figure 1A**. We investigated whether GLYX-13 attenuates MK-801-induced hyperlocomotion in the OFT. As shown in **Figure 1B**, the two-way ANOVA revealed significant differences for drug treatment [*F*(4,330) = 72.6, *P* < 0.001] and time [*F*(5,330) = 25.09, *P* < 0.0001] in the distances traveled during the 5-min intervals. However, there was no significant difference for treatment × time interaction [*F*(20,330) = 1.514, *P* = 0.0740]. *Post hoc* analysis showed that GLYX-13 (0.5 and 1 mg/kg) significantly reversed hyperlocomotion induced by MK-801. Additionally, the one-way ANOVA revealed a significant treatment effect on the total distance traveled in the OFT [*F*(4,55) = 387.2, *P* < 0.0001]. As shown in **Figure 1C**, the repeated administration of MK-801 significantly increased the distance traveled compared with vehicle 1- and 2-treated controls in the OFT (*P* < 0.001). However, MK-801-induced hyperlocomotion was significantly attenuated by treatment with GLYX-13 (0.5 mg/kg, *P* < 0.001 and 1 mg/kg, *P* < 0.001).

### GLYX-13 Significantly Reversed Repeated Treatment With MK-801

**FIGURE 2:**
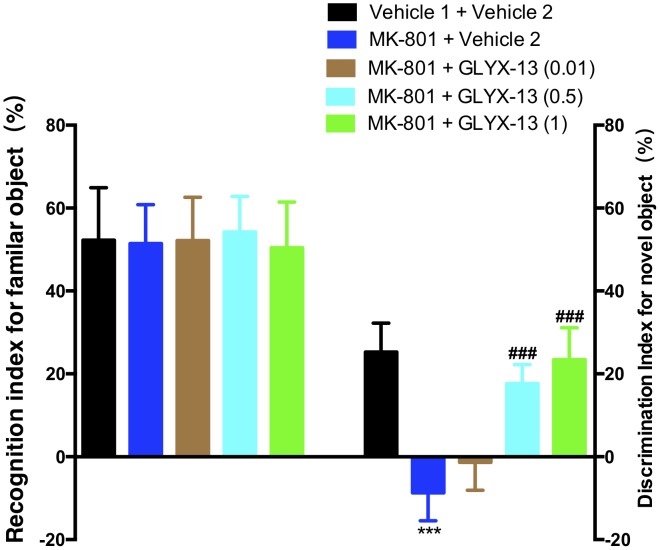
Effects of GLYX-13 on MK-801-induced object recognition deficits in mice through NORT. The recognition index of the familiar objects and the discrimination index of the novel object are shown. Data represent the mean ± SEM (*n* = 12 per group; ^∗∗∗^*P* < 0.001, versus the vehicle 1 + vehicle 2-treated group; ^###^*P* < 0.001, versus the MK-801 + vehicle 2-treated group).

**FIGURE 3:**
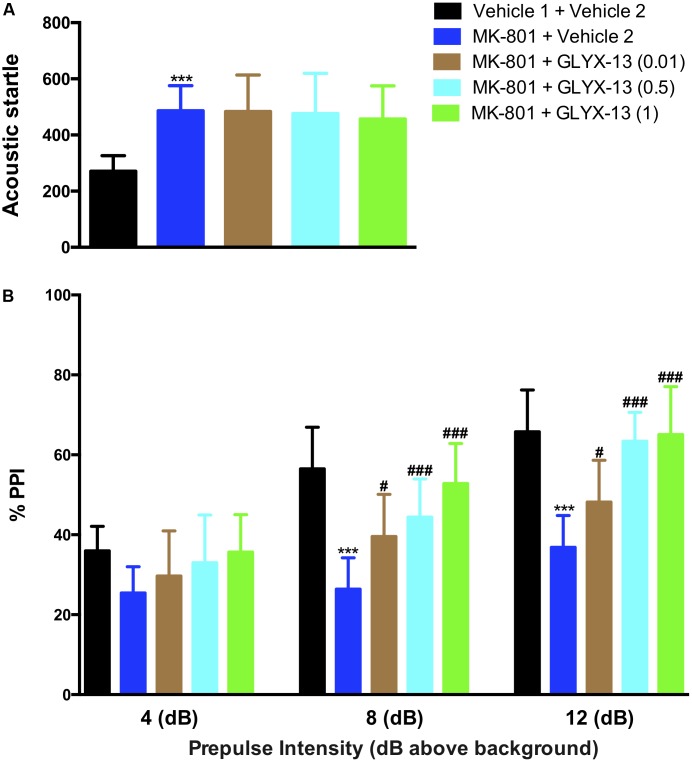
GLYX-13 significantly attenuated MK-801-induced impairments in PPI but not the startle response. **(A)** MK-801 significantly increased the startle amplitude. **(B)** Effect of GLYX-13 on PPI inhibition of the startle response at different prepulses (4, 8, and 12 dB above background) presented before the startle pulse intensity (120 dB). Data represent the mean ± SEM (*n* = 12 per group; ^∗∗∗^*P* < 0.001, versus the vehicle 1 + vehicle 2-treated group; ^#^*P* < 0.05 and ^###^*P* < 0.001, versus the MK-801 + vehicle 2-treated group).

As shown in the left panel of **Figure 2**, the recognition index for the familiar object in the acquisition trial showed no significant difference in any of the groups [*F*(4,55) = 0.2157, *P* = 0.9286]. One-way ANOVA indicated a significant difference in the discrimination index of the novel object among all groups [*F*(4,55) = 64.21, *P* < 0.0001]. Further *post hoc* analysis revealed that the MK-801 + vehicle 2 group showed a significant decrease in the preference for the novel object compared with the vehicle 1 + vehicle 2 group (*P* < 0.001). However, this effect was significantly abolished in mice treated with GLYX-13 (0.5 mg/kg, *P* < 0.001 and 1 mg/kg, *P* < 0.001). No significant effect was observed among all groups in total exploration times (acquisition trial + retention trial, data not shown). We evaluated whether GLYX-13 ameliorates the MK-801-induced sensorimotor-gating deficits, including startle enhancement in the acoustic startle response and PPI disruption. We found that the repeated administration of MK-801 alone (0.5 mg/kg) significantly increased the acoustic startle amplitude at 120 dB [*F*(4,55) = 8.232, *P* < 0.0001, **Figure 3A**] and decreased PPI at 4 dB [*F*(4,55) = 2.707, *P* = 0.0394, **Figure 3B**], 8 dB [*F*(4,55) = 17.90, *P* < 0.0001, **Figure 3B**], and 12 dB [*F*(4,55) = 20.74, *P* < 0.0001, **Figure 3B**] above the background noise, which indicates MK-801-induced sensorimotor-gating deficits. Startle amplitude enhancement induced by MK-801 was not reversed with three doses of GLYX-13. However, PPI disruption induced by MK-801 was significantly prevented by the administration of GLYX-13 (0.5 or 1 mg/kg) in a dose-dependent manner.

### Effects of GLYX-13 on NR2B and DISC1 Expression Levels

**FIGURE 4:**
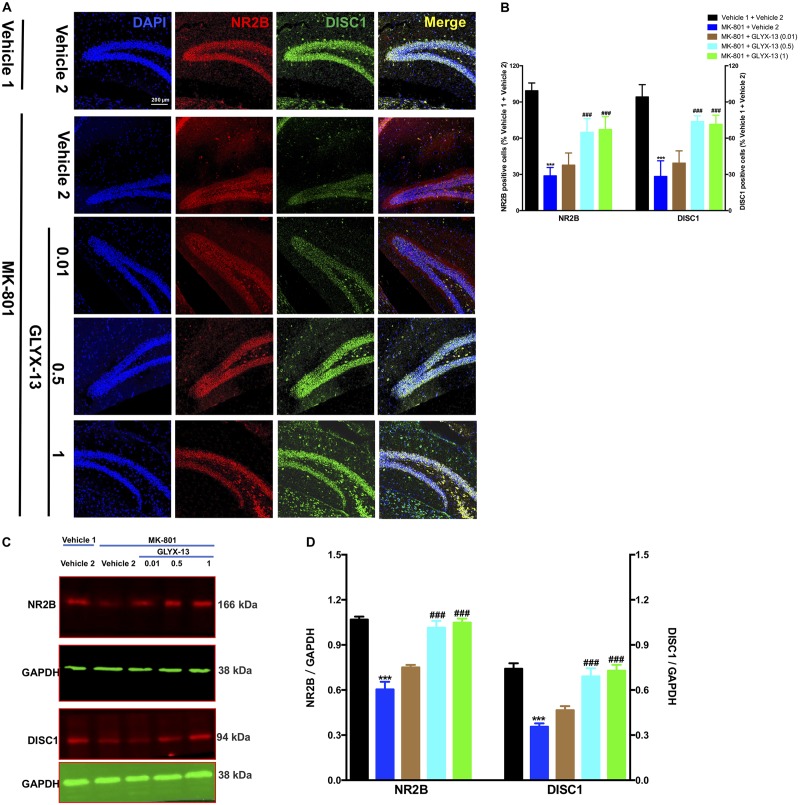
GLYX-13 restored the decrease in NR2B and DISC1 expressions in the dentate gyrus. **(A)** Confocal microscopy images show double staining for NR2B (red) or DISC1 (green) and merged images in hippocampal slices. Scale bar = 200 μm. **(B)** Quantitative analyses of NR2B and DISC1-immunopositive cells in the DG. **(C,D)** Representative images of immunoblots using antibodies against NR2B and DISC1 along with quantitative analyses. Data are expressed as the mean ± SEM (*n* = 6 per group for immunofluorescent analysis and *n* = 3 per group for western blotting; ^∗∗∗^*P* < 0.001 compared with the vehicle 1 + vehicle 2 group; ^###^*P* < 0.001 compared with the MK-801 + vehicle 2 group).

We investigated whether GLYX-13 affects decreased expression levels of NR2B and DISC1 induced by MK-801 in the hippocampal tissue of mice. To identify NR2B- and DISC1-immunopositive cells, hippocampal slices were double-stained with antibodies against NR2B and DISC1. As shown in **Figure 4A**, confocal microscopy images reveal double staining for NR2B (red) and DISC1 (green) in hippocampal slices from all treatment groups. Compared with the vehicle 1 + vehicle 2 group (**Figure 4B**), the number of NR2B- [*F*(4,25) = 53.54, *P* < 0.001] and DISC1- [*F*(4,25) = 48.09, *P* < 0.001] positive neuronal cells significantly decreased with MK-801. However, treatment with GLYX-13 at 0.5 mg/kg (*P* < 0.001) and 1 mg/kg (*P* < 0.001), but not at 0.01 mg/kg, for 7 days significantly increased the number of NR2B- and DISC1-immunopositive cells compared with the MK-801 + vehicle 2 group. Additionally, we found that the administration of MK-801 significantly decreased protein expression levels of NR2B [*F*(4,10) = 36.00, *P* < 0.001] and DISC1 [*F*(4,10) = 22.02, *P* < 0.001] in the hippocampus (**Figures 4C,D**), which were attenuated by the administration of GLYX-13 (NR2B: 0.5 mg/kg and 1 mg/kg, both *P* < 0.001; DISC1: 0.5 mg/kg, *P* < 0.01, and 1 mg/kg, *P* < 0.001). The western blotting antibodies for NR2B (first antibody: Abcam, Cat#: ab28373; second antibody: Thermo Fisher Scientific, Cat#: A21036) and for DISC1 (first antibody: Abcam, Cat#: ab192258; second antibody: Thermo Fisher Scientific, Cat#: A21038) were used. The access to the analyzed blots were shown in the Supplementary Data.

### NR2B Knockdown Did Not Induce Hyperlocomotion

**FIGURE 5:**
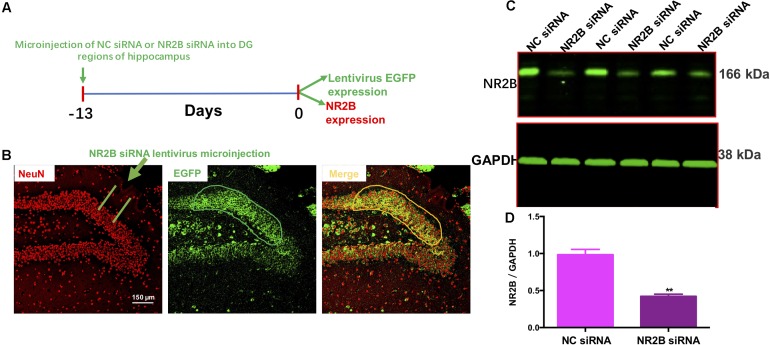
Lentivirus-mediated NR2B knock-down in the dentate gyrus. **(A)** Experimental procedure for the test schedule. NC siRNA or NR2B siRNA were microinjected into the hippocampus following a 13-day acclimatization. **(B)** NC siRNA or NR2B siRNA were well-expressed in the hippocampal DG regions as indicated by the merged images, which include nuclear staining with DAPI (blue) and green fluorescent protein (GFP, green) observed under a fluorescence microscope. Scale bars = 200 μm. **(C,D)** The expression of NR2B was significantly decreased by NR2B siRNA in the hippocampus of mice. Data are expressed as means ± SEM (*n* = 3; ^∗∗^*P* < 0.01 compared with NC siRNA group).

To examine the effects of the NR2B siRNA lentivirus, NC siRNA or NR2B siRNA were microinjected into the hippocampal DG regions following a 13-day recovery (**Figure 5A**). Fluorescent microscopy showed that NR2B siRNA were well-expressed in the neurons of the DG regions, as indicated by EGFP-positive cells (green) (**Figure 5B**). Additionally, NR2B protein expression in the hippocampus significantly decreased (*t* = 7.327, df = 4, *P* < 0.01, **Figures 5C,D**) compared with the NC siRNA group, indicating the knockdown effects of NR2B siRNA in mice. The western blotting antibodies for NR2B (first antibody: Abcam, Cat#: ab28373; second antibody: Thermo Fisher Scientific, Cat#: A32723) were used. The access to the analyzed blots were shown in the Supplementary Data.

**FIGURE 6:**
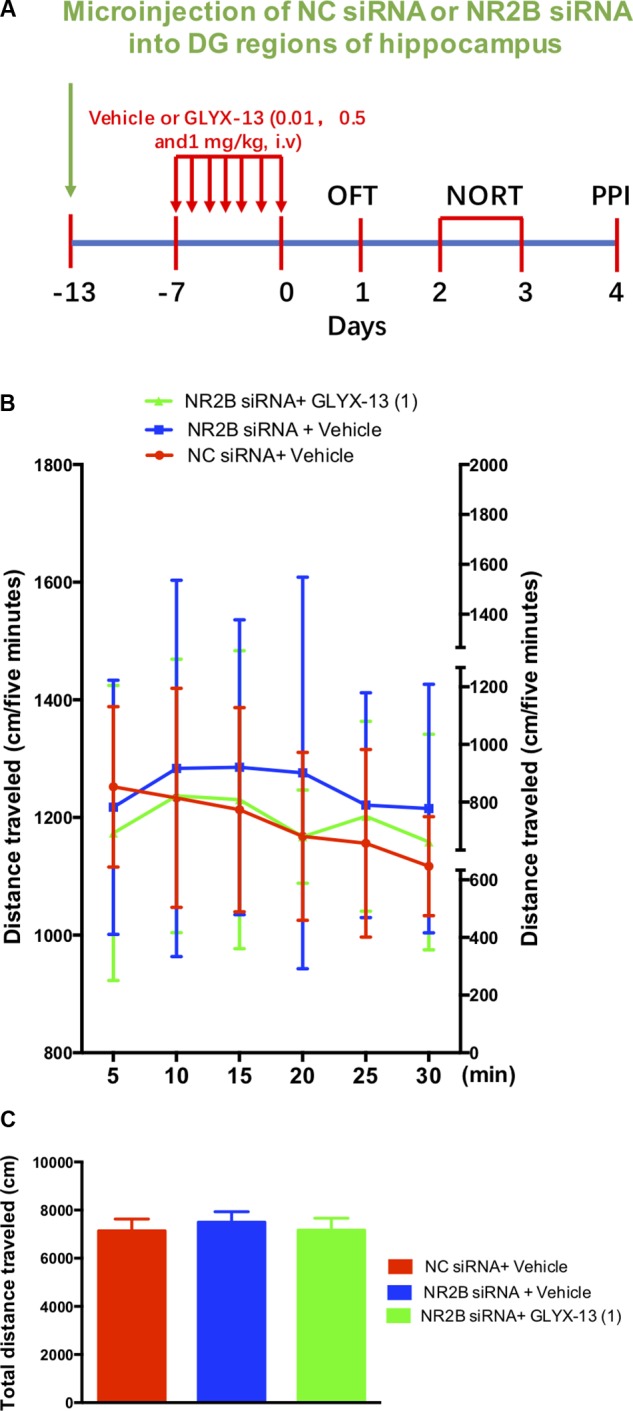
GLYX-13 did not change the locomotor activity NR2B siRNA knockdown mice. **(A)** Experimental time line. **(B)** Horizontal locomotor activity was examined as the distance traveled (cm) in 30 min. The distances traveled in 5 min intervals **(B)** and the total distance traveled in 30 min **(C)** are shown. Data are expressed as means ± SEM (*n* = 12).

**FIGURE 7:**
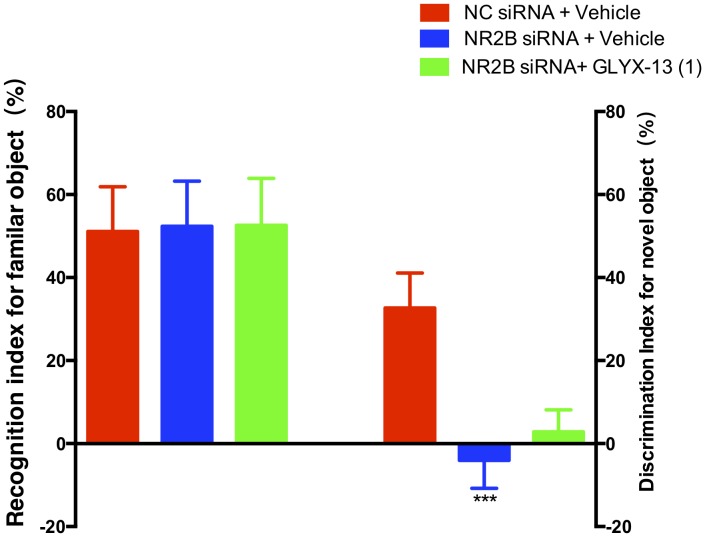
GLYX-13 did not reverse the object recognition deficits induced by NR2B siRNA in the mice during the NORT. The recognition index of the familiar objects and the discrimination index for the novel object are shown. Data represent the mean ± SEM (*n* = 12 per group; ^∗∗∗^*P* < 0.001, versus the NC siRNA + vehicle-treated group).

Based on these findings, we clarified whether NR2B is involved in schizophrenia-like behaviors and alleviated by GLYX-13 in mice. Our experimental design is shown in **Figure 6A**. The OFT was conducted before the other behavioral tests to explore whether the NR2B siRNA-mediated downregulation of NR2B in the hippocampus produces alterations in locomotor activity in mice. All treatments had no effects on the distances traveled in 5-min intervals [*F*(2,198) = 1.817, *P* = 0.1652, **Figure 6B**] and the total distance traveled during 30 min [*F*(2,33) = 2.121, *P* = 0.1360, **Figure 6C**] in the OFT. Following the locomotor activity test, mice were subjected to NORT and PPI. All treatments had no significant effect on the recognition index for the familiar objects in the training trial [*F*(2,33) = 0.06403, *P* = 0.9381, **Figure 7**, left panel], indicating a similar preference for each of the two identical objects. One-way ANOVA showed significant group effects in the discrimination index of the novel object in the test trial of the NORT [*F*(2,33) = 95.17, *P* < 0.0001, **Figure 7**, right panel]. *Post hoc* tests revealed that NR2B knockdown mice spent less discrimination index on the novel object in the test trial, which was significantly lower compared with the NC siRNA + vehicle treated control group (*P* < 0.001). The low percentage of exploration time for the novel object induced by NR2B knockdown was not significantly reversed with the administration of GLYX-13 (1 mg/kg, *P* > 0.05).

### NR2B Knockdown Blocked the Alleviating Effects of GLYX-13

**FIGURE 8:**
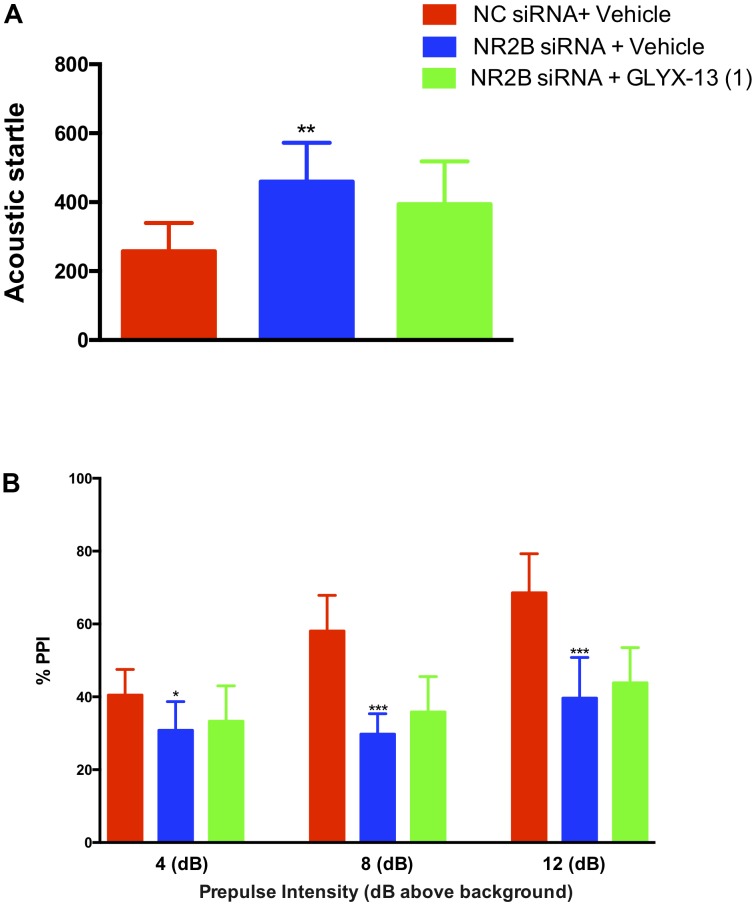
GLYX-13 did not attenuate the NR2B siRNA-induced impairments in PPI of mice. **(A)** NR2B siRNA significantly increased the startle amplitude. **(B)** Effect of GLYX-13 on PPI inhibition induced by NR2B siRNA in mice. Data represent the mean ± SEM (*n* = 12 per group; ^∗^*P* < 0.05, ^∗∗^*P* < 0.01, and ^∗∗∗^*P* < 0.001, versus the NC siRNA + vehicle-treated group).

In this study, we confirmed that NR2B knockdown in the hippocampus induces sensorimotor-gating deficits and GLYX-13 ameliorates the NR2B siRNA-induced acoustic startle response and PPI disruption in mice. We found that the knockdown of NR2B in the hippocampus significantly increased the acoustic startle amplitude at 120 dB [*F*(2,33) = 10.99, *P* < 0.01, **Figure 8A**], as well as decreased the PPI at 4 dB [*F*(2,33) = 4.420, *P* < 0.05], 8 dB [*F*(2,33) = 36.04, *P* < 0.0001], and 12 dB [*F*(2,33) = 26.44, *P* < 0.0001, **Figure 8B**] above the background noise, indicating NR2B siRNA-induced sensorimotor gating deficits. GLYX-13 did not reverse startle amplitude enhancement induced by NR2B siRNA. Additionally, GLYX-13 (1 mg/kg) administration did not significantly alleviate PPI disruption induced by NR2B siRNA, suggesting that NR2B acts as a critical mediator in the anti-schizophrenia-like effects of GLYX-13.

### NR2B Knockdown Downregulates DISC1 and Blocks Upregulation of DISC1 by GLYX-13

**FIGURE 9:**
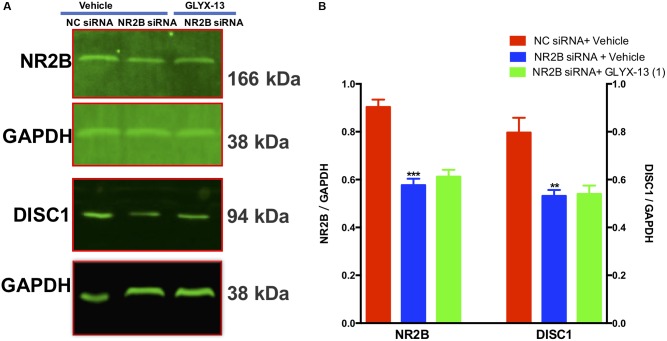
GLYX-13 did not reverse the downregulation of DISC1 induced by NR2B siRNA in mice. **(A,B)** Representative images of immunoblots using antibodies against NR2B and DISC1 along with quantitative analyses. Data are expressed as the mean ± SEM (*n* = 4 per group for western blotting; ^∗∗^*P* < 0.01 and ^∗∗∗^*P* < 0.001 compared with the NC siRNA + vehicle-treated group).

Our results showed that NR2B siRNA significantly decreased NR2B protein expression in the DG [*F*(2,9) = 38.71, *P* < 0.001, **Figures 9A,B**]. However, GLYX-13 did not reverse this loss. Additionally, the NR2B knockdown significantly decreased DISC1 protein expression levels [*F*(2,9) = 12.12, *P* < 0.01, **Figures 9A,B**] in the hippocampus. However, the downregulation of DISC1 was not attenuated by the administration of GLYX-13. The western blotting antibodies for NR2B (first antibody: Abcam, Cat#: ab28373; second antibody: Thermo Fisher Scientific, Cat#: A32723) and for DISC1 (first antibody: Abcam, Cat#: ab192258; second antibody: Thermo Fisher Scientific, Cat#: A32735) were used. The access to the analyzed blots were shown in the Supplementary Data.

## Discussion

It has been well-established that NMDAR play a key role in the etiology and pharmacological treatments of schizophrenia (Gilmour et al., 2012; Jadi et al., 2016; Dauvermann et al., 2017; Jeon et al., 2017). For instance, a considerable amount of research has demonstrated that NMDAR antagonists mimic psychosis-relevant behaviors and deficits in sensory gating (Jeon et al., 2017; Oh et al., 2017). Based on this hypothesis, the current study evaluated the involvement of NMDAR subunits in the symptoms of schizophrenia in mice induced by the repeated administration of the NMDAR antagonist MK-801. Additionally, we investigated whether the NMDAR partial agonist GLYX-13 alleviates hyperlocomotion, memory, and sensorimotor-gating deficits (as measured by PPI) induced by MK-801.

GLYX-13 is a tetrapeptide derived from a monoclonal antibody that acts as an NMDAR functional glycine-site modulator and cognitive enhancer (Moskal et al., 2005, 2014; Rodriguez et al., 2016). It is possible that GLYX-13 exerts an alleviating effect on psychosis-relevant behaviors induced by MK-801 through the modulation of NMDAR. Hyperlocomotion, a positive symptom of schizophrenia, is induced by MK-801 in rodent models (Kruk-Slomka et al., 2017; Gould et al., 2018). Consistent with previous reports (Gomes et al., 2014; Park et al., 2014; Nishiyama et al., 2017), we found that the repeated administration of MK-801 induced hyperlocomotion in the OFT of mice. Notably, to the best of our knowledge, this study is the first to show that hyperlocomotion induced by MK-801 was significantly alleviated by GLYX-13 in mice, suggesting that GLYX-13 ameliorates the positive symptoms of schizophrenia. Additionally, previous behavioral testing has identified impairments in novel object recognition memory as a key feature in chronic NMDAR inhibitor-induced schizophrenia-like behaviors in rodent models (Rajagopal et al., 2014). Our results confirmed that chronic treatment with MK-801 caused impairments in novelty exploration and recognition, which might represent both motivational and cognitive symptoms of schizophrenia. In agreement with our hypothesis, we observed that MK-801-induced recognition memory impairments that were attenuated by GLYX-13. Our results are consistent with those of a recent study that demonstrated that the NMDAR inhibitor ketamine induced novel object recognition deficits that were significantly reversed with GLYX-13 in mice (Rajagopal et al., 2016). Therefore, these results further indicate that GLYX-13 may improve cognition in schizophrenia rodent models. Furthermore, PPI disruption is an indicator of MK-801-induced core symptoms of schizophrenia in rodents (Khella et al., 2014; Burrows et al., 2015), specifically a disruption in sensorimotor gating (Braff et al., 2001). Our present study showed that repeated administration of MK-801 induced PPI deficits in mice, indicating a disruption of sensorimotor gating. Notably, MK-801-induced PPI deficits were markedly reversed by the administration of GLYX-13, although the startle enhancement induced by MK-801 remain unchanged.

Several studies have shown that the hippocampus plays a critical role in cognitive functions disrupted in schizophrenia because of changes in NMDAR, implicating the hippocampus in the pathophysiology of schizophrenia (Sweatt, 2004; Nomura et al., 2016). We focused on the molecular mechanisms of GLYX-13 in schizophrenia-like behaviors induced by MK-801 in the hippocampus. From a molecular perspective, NMDAR are tetrameric structures assembled from two obligatory GluN1 subunits and two GluN2A (formerly NR2A) or GluN2B (formerly NR2B) subunits. Evidence suggests that the NR2B subunit in the hippocampus is particularly important for NMDAR channel function (Akashi et al., 2009), long-term potentiation (LTP), and associated cognitive functions such as spatial learning (Clayton et al., 2002; Wang et al., 2017; Xu et al., 2017). Accordingly, numerous studies have suggested that NR2B, rather than NR2A, is involved in schizophrenia in humans (Loftis and Janowsky, 2003; Geddes et al., 2014) and schizophrenia-like behaviors induced by NMDAR antagonists in rodents (Jiménez-Sánchez et al., 2014). Our results are consistent with the molecular changes observed in the hippocampus of schizophrenia patients (Geddes et al., 2014), namely the expression of NR2B in the hippocampus of mice was significantly decreased by MK-801. Notably, GLYX-13 significantly reversed this downregulation of NR2B in the hippocampus, indicating that the downregulation of the NR2B subunits sufficiently elicit psychotomimetic activity induced by MK-801. However, the contribution of the NR2B subunit to the biochemical and behavioral changes elicited by NMDAR antagonists is still poorly understood.

Many biochemical, molecular, and pharmacological studies have demonstrated the functional interactions between DISC1 (a risk factor for schizophrenia) and NMDAR (Namba et al., 2011; Ramsey et al., 2011; Snyder and Gao, 2013; Wu and Barger, 2016). Our results revealed that DISC1 expression was significantly decreased by MK-801 in the hippocampus of mice. This is in concordance with a previous study that demonstrated that DISC1 protein levels are regulated by NMDAR signaling (Ramsey et al., 2011), suggesting that DISC1 functional activity is downstream to NMDAR signaling. Consistent with our expectations, the downregulation of DISC1 was markedly reversed by GLYX-13 in mice. However, it remains unknown whether the antipsychotic effects of GLYX-13 are a result of the increased levels of DISC1 in the hippocampus of mice. Therefore, we further investigated whether NR2B-mediated DISC1 signaling is involved in the antipsychotic effects of GLYX-13 in mice.

Previous studies have revealed that interference with the expression and/or function of the NR2B subunit produces deficits in synaptic plasticity and memory in rodents (Duffy et al., 2008; Brigman et al., 2010). In the present study, we found that novel object recognition ability and PPI disruption were significantly decreased by siRNA-mediated NR2B knockdown in the hippocampus of mice. Additionally, DISC1 was also downregulated in the NR2B knockdown, suggesting that NR2B subunit composition and DISC1 downregulation in the hippocampus contribute to the pathophysiology of schizophrenia. In concordance, positive allosteric modulation selective for NR2B has been proposed as a novel therapeutic target for the treatment of schizophrenia and cognitive dysfunction (Mony et al., 2009; Menniti et al., 2013). Our results revealed that NR2B knockdown significantly abolished the effects of GLYX-13 on memory deficits, PPI disruption, and downregulation of NR2B and DISC1 induced by NR2B knockdown in mouse hippocampus. Our findings further support the idea that NR2B and DISC1 in the hippocampus are involved in the effects of GLYX-13 and are potentially therapeutic for cognitive and PPI dysfunction in schizophrenia. However, in contrast with previous studies that have revealed that NR2B antagonists produce hyperlocomotor activity in rodents (Chaperon et al., 2003; Mathur et al., 2009), the present study revealed that a single knockdown of the NR2B subunit in the hippocampus did not induce hyperlocomotion in mice, possibly indicating that the hippocampal-restricted deletion of NR2B could not induce the positive symptoms of schizophrenia in mice. We cannot exclude the possibility of other molecular mechanisms underlying the decrease in positive symptoms (hyperlocomotion) induced by MK-801 in mice. Additionally, surmounting evidence links NMDAR hypofunction in the prefrontal cortex (PFC) as an underlying pathological origin of psychiatric disorders (Monaco et al., 2015), the involvement of PFC in the alleviating effects of GLYX-13 on the schizophrenia should be conducted in the future study.

## Conclusion

The present study revealed that GLYX-13 may be a promising drug for deficits in learning and PPI associated with schizophrenia. We further demonstrated that the downregulation of NR2B and DISC1 in the hippocampus may be associated with cognitive dysfunction and PPI symptoms in schizophrenia rodent models.

## Author Contributions

CW and ZC designed the study. DZ, DL, ZW, and YZ performed the experiments and drafted the manuscript. CW wrote the first draft of the manuscript, which all other authors reviewed. All the authors approved publication.

## Conflict of Interest Statement

The authors declare that the research was conducted in the absence of any commercial or financial relationships that could be construed as a potential conflict of interest.
