# Supplementary material for: GLYX-13 Ameliorates Schizophrenia-Like Phenotype Induced by MK-801 in Mice: Role of Hippocampal NR2B and DISC1
Source: Front Mol Neurosci. 2018 Apr 11;11:121. doi: 10.3389/fnmol.2018.00121 (PMC5904356; doi:10.3389/fnmol.2018.00121)
Supplement: Supplementary file 1 [file Presentation_1.PDF]

GLYX-13 ameliorates schizophrenia-like phenotype induced by MK-801 in mice: Role of hippocampal NR2B and DISC1

Dongsheng Zhou[1,2]△, Dan Lv[2,3,4]△, Zhen Wang[5]△, Yanhua Zhang[2,3,4], Zhongming Chen[1*], Chuang Wang[2,3,4*]

1. Ningbo Kangning Hospital, Ningbo, Zhejiang 315201, China
2. Ningbo Key Laboratory of Behavioral Neuroscience, Ningbo University School of Medicine, 818 Fenghua Road, Ningbo, Zhejiang 315211, China
3. Zhejiang Provincial Key Laboratory of Pathophysiology, Ningbo University School of Medicine, 818 Fenghua Road, Ningbo, Zhejiang 315211, China
4. Department of Physiology and Pharmacology, Ningbo University School of Medicine, 818 Fenghua Road, Ningbo, Zhejiang 315211, China
5. CAS Key Laboratory for Receptor Research, Shanghai Institute of Materia Medica, Chinese Academy of Sciences, Shanghai 201203, China

△These authors contributed equally to this study.

***Correspondence:**

Chuang Wang, MD, PhD

Ningbo Key Laboratory of Behavioral Neuroscience

Zhejiang Provincial Key Laboratory of Pathophysiology

Department of Physiology and Pharmacology

Ningbo University School of Medicine

Ningbo, Zhejiang 315211, PR China

E-mail: wanglovechuang@163.com; wangchuang@nbu.edu.cn

Zhongming Chen, MD

Ningbo Kangning Hospital

Ningbo, Zhejiang 315210, PR China

E-mail: chenzhongming@hotmail.com

Analysis of the western blotting data

NR2B

GAPDH

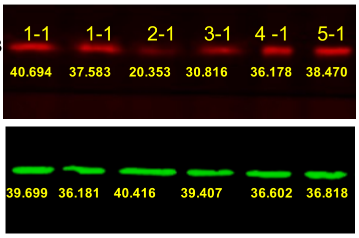

NR2B/GAPDH 1.0387 0.5036 0.7819 0.9884 1.0449

NR2B

GAPDH

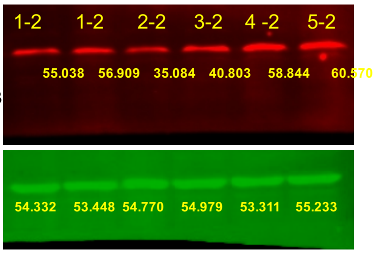

NR2B/GAPDH 1.0648 0.6406 0.7422 1.1038 1.0967

NR2B

GAPDH

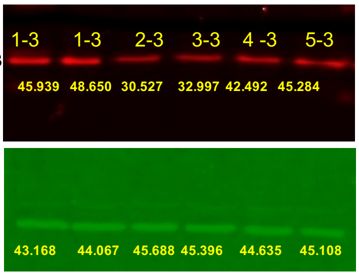

NR2B/GAPDH 1.1040 0.6682 0.7269 0.9520 1.0040

1-1, 1-2 and 1-3: Number 1, 2 and 3 of the **Vehicle 1 + Vehicle 2 group**
2-1, 2-2 and 2-3: Number 1, 2 and 3 of the **MK-801 + Vehicle 2 group**
3-1, 3-2 and 3-3: Number 1, 2 and 3 of the **MK-801 + GLYX-13 (0.01) group**
4-1, 4-2 and 4-3: Number 1, 2 and 3 of the **MK-801 + GLYX-13 (0.5) group**
5-1, 5-2 and 5-3: Number 1, 2 and 3 of the **MK-801 + GLYX-13 (1) group**

F (DFn, DFd)	P value
F (4, 10) = 36.00	P < 0.0001

DISC1

GAPDH

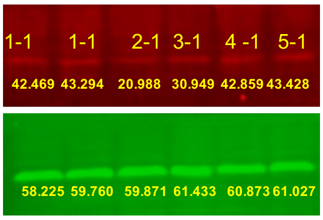

DISC1/GAPDH 0.7245 0.3506 0.5037 0.7041 0.7116

DISC1

GAPDH

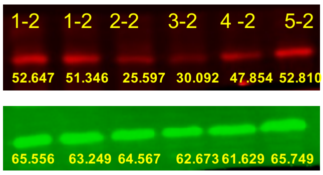

DISC1/GAPDH 0.8118 0.3964 0.4801 0.7765 0.8032

DISC1

GAPDH

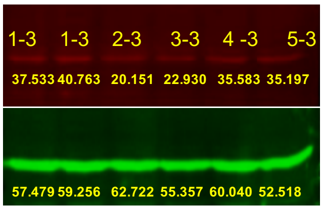

DISC1/GAPDH 0.6880 0.3213 0.4142 0.5930 0.6702

1-1, 1-2 and 1-3: Number 1, 2 and 3 of the **Vehicle 1 + Vehicle 2 group**
2-1, 2-2 and 2-3: Number 1, 2 and 3 of the **MK-801 + Vehicle 2 group**
3-1, 3-2 and 3-3: Number 1, 2 and 3 of the **MK-801 + GLYX-13 (0.01) group**
4-1, 4-2 and 4-3: Number 1, 2 and 3 of the **MK-801 + GLYX-13 (0.5) group**
5-1, 5-2 and 5-3: Number 1, 2 and 3 of the **MK-801 + GLYX-13 (1) group**

F (DFn, DFd)	P value
F (4, 10) = 22.02	P < 0.0001

NR2B

GAPDH

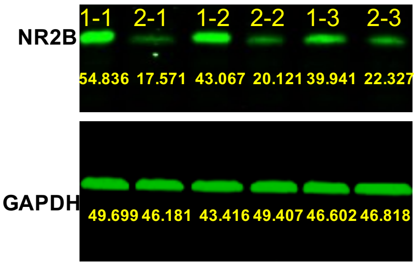

NR2B/GAPDH 1.1034 0.3804 0.9920 0.4072 0.8571 0.4769

1-1, 1-2 and 1-3: Number 1, 2 and 3 of the **NC siRNA group**
2-1, 2-2 and 2-3: Number 1, 2 and 3 of the **NR2B siRNA group**

P value	0.0018
P value summary	**
Significantly different? (P < 0.05)	Yes
One- or two-tailed P value?	Two-tailed
t, df	t=7.327 df=4

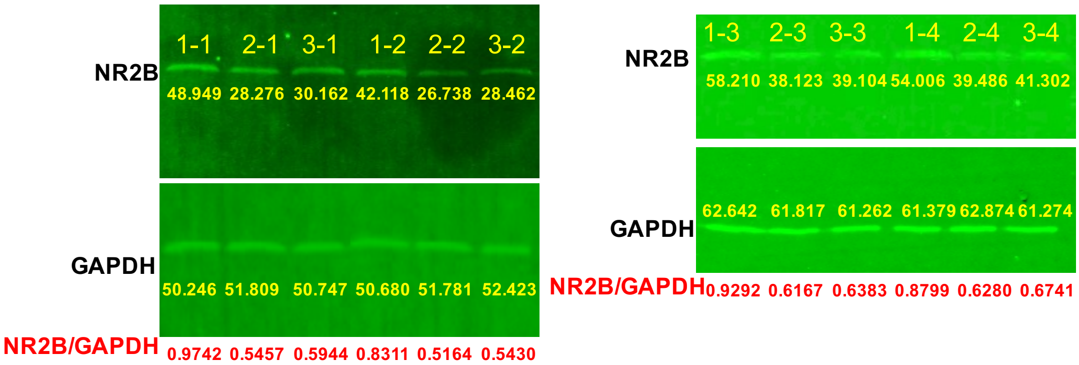

1-1, 1-2, 1-3 and 1-4: Number 1, 2, 3 and 4 of the **NC siRNA + Vehicle group**
2-1, 2-2, 2-3 and 2-4: Number 1, 2, 3 and 4 of the **NR2B siRNA + Vehicle group**
3-1, 3-2, 3-3 and 3-4: Number 1, 2, 3 and 4 of the **NR2B siRNA + GLYX-13 (1) group**

F (DFn, DFd)	P value
F (2, 9) = 38.71	P < 0.0001

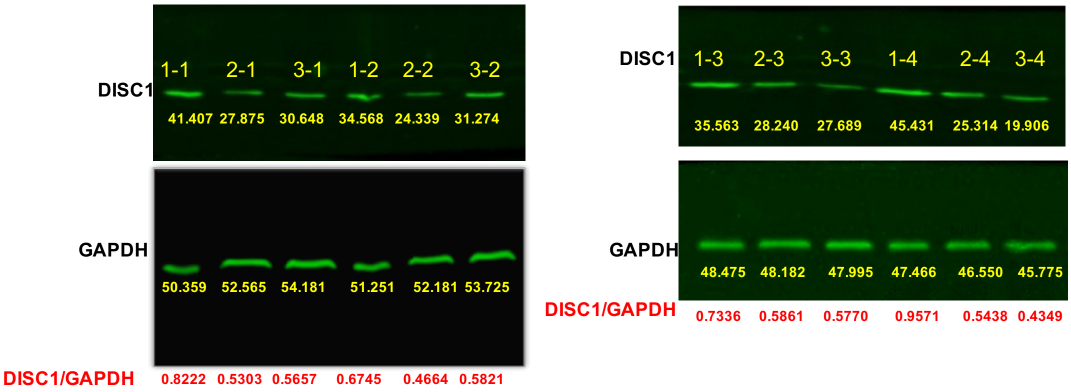

1-1, 1-2, 1-3 and 1-4: Number 1, 2, 3 and 4 of the **NC siRNA + Vehicle group**
2-1, 2-2, 2-3 and 2-4: Number 1, 2, 3 and 4 of the **NR2B siRNA + Vehicle group**
3-1, 3-2, 3-3 and 3-4: Number 1, 2, 3 and 4 of the **NR2B siRNA + GLYX-13 (1) group**

F (DFn, DFd)	P value
F (2, 9) = 12.12	P = 0.0028
